# Artificial Intelligence Use and Views: Key Findings From the National Poll on Healthy Aging

**DOI:** 10.1093/geroni/igaf122.4314

**Published:** 2025-12-31

**Authors:** Erica Solway, Robin Brewer, J Scott Roberts, Dianne Singer, Sydney Strunk, Nicholas Box, Matthias Kirch

**Affiliations:** University of Michigan, Ann Arbor, Michigan, United States; University of Michigan, Ann Arbor, Michigan, United States; University of Michigan School of Public Health, Ann Arbor, Michigan, United States; University of Michigan, Ann Arbor, Michigan, United States; University of Michigan, Ann Arbor, Michigan, United States; University of Michigan, Ann Arbor, Michigan, United States; University of Michigan, Ann Arbor, Michigan, United States

## Abstract

The spread of artificial intelligence (AI) technologies offers opportunities to improve home safety, provide health information, and support aging in place, but it also raises concerns about risks to health and well-being. Little is known about older adults’ use and views on AI related to healthy aging. In February 2025, the University of Michigan National Poll on Healthy Aging surveyed a diverse sample of 2,883 adults age 50 and older about their experiences with and views on AI technologies. Overall, 55% of adults age 50 and older said they had ever used AI technologies that one speaks or types to, with 14% using AI for health information. Many reported benefits of using AI-powered devices, including voice assistants and home security devices and systems, for aging in place. Overall, 35% of adults age 50 and older reported interest in using AI in their day-to-day lives. At the same time, 92% of adults age 50 and older agreed they want to know if the information they receive is from a person or from AI, and 81% wanted to learn more about the risks of AI. Nearly half (46%) had very little or no trust in AI-generated health information. This presentation will describe notable differences in AI use and perspectives by demographic subgroups and will conclude with a discussion of how these findings can be used for the development of programs to assist older adults in understanding the risks of AI and in safely and effectively using AI to support healthy aging.

